# Improving BLE-Based Localization in Multipath-Affected Indoor Environments

**DOI:** 10.3390/s26216767

**Published:** 2026-10-26

**Authors:** Shawana Jamil, Dae-Ho Kim, Jae-Young Pyun

**Affiliations:** 1 Department of Information and Communication Engineering, Chosun University, Gwangju 61452, Republic of Korea; shawana@chosun.ac.kr; 2 Department of Artificial Intelligence Engineering, Chosun University, Gwangju 61452, Republic of Korea; wireless@chosun.ac.kr

**Keywords:** BLE-based localization, LOS/NLOS classification, adaptive Kalman filter, weighted centroid localization, ensemble learning, multipath-affected indoor environments

## Abstract

Received signal strength indicator (RSSI)-based positioning with Bluetooth Low Energy (BLE) is widely used for indoor localization, and weighted centroid localization (WCL) is among the most common methods because of its simplicity and low computational cost. However, BLE RSSI is subject to temporal fluctuation and multipath interference, which induce an unstable RSSI-to-distance relationship and degrade the accuracy of conventional WCL. Its distance-based weighting cannot distinguish reliable line-of-sight (LOS) anchors from misleadingly strong non-line-of-sight (NLOS) anchors. This limitation is most severe in long, anchor-dense corridors and high-traffic lobbies, where multipath propagation is high. This paper presents a confidence-adaptive Kalman filtering framework for BLE RSSI-based localization in multipath-affected environments. A scan-level confidence score combines relative RSSI ranking, probabilistic LOS/PLOS/NLOS classification, and the proportion of high-confidence LOS predictions. The resulting score adapts the measurement covariance assigned to WCL position observations without modifying individual anchor weights. Relative to conventional WCL, the proposed method reduced the mean absolute error by 14.6% in corridors, 9.3% in a classroom, 3.3% in an office, and 7.9% along a high-traffic lobby trajectory. Relative to a fixed-covariance Kalman filter, the additional reduction was small in the low-traffic zones (0.4–2.0%) and largest in the high-traffic lobby (7.7%).

## 1. Introduction

Indoor localization using wireless signals such as Wi-Fi and Bluetooth Low Energy (BLE) has gained widespread popularity due to its low cost, compatibility with mobile devices, wide availability, and effectiveness in global navigation satellite system (GNSS)-inaccessible environments. Applications include indoor navigation in large hospitals, shopping malls, underground subways, university campuses, and patient monitoring in smart healthcare systems [1]. Among the available navigation technologies, BLE localization has emerged as a cost-effective and smartphone-based indoor positioning solution [2,3] using trilateration [4], triangulation [5], and weighted centroid localization (WCL) [6]. WCL remains widely adopted due to its simplicity, low computational overhead, and minimal training requirements, making it suitable for resource-constrained systems. This localization method operates under the assumption that a stronger received signal strength indicates closer proximity and higher reliability. However, this assumption often fails in real-world indoor environments due to signal propagation effects such as attenuation, multipath propagation, and obstructions [7]. In some such cases, non-line-of-sight (NLOS) signals can appear stronger than actual line-of-sight (LOS) signals, leading to significant localization errors [5,8,9].

Ranging technologies such as Wi-Fi round-trip time (RTT) and ultra-wideband (UWB) provide higher accuracy, but they depend on client- and infrastructure-side hardware and firmware support that is not uniformly available across the installed device base, and they still require NLOS handling [9,10,11]. Visible-light positioning can reach centimeter-level accuracy even with sparse RSS fingerprints [12]; however, it requires modulated LED luminaires and an optical line of sight to the receiver. Received signal strength indicator (RSSI), in contrast, is available on every BLE-capable consumer device and existing beacon infrastructure without ranging hardware, fingerprint surveys, or significant energy cost. The question addressed in this work is therefore not whether RSSI can match ranging accuracy, but how to obtain more reliable position estimates from unstable BLE RSSI under multipath propagation without changing the infrastructure.

This degradation is most severe in large, anchor-dense indoor deployments such as long corridors and high-traffic lobbies, where multipath propagation is pronounced and labor-intensive fingerprint maps are impractical to construct and maintain. This work is scoped to BLE-based localization using commodity RSSI measurements and without a pre-surveyed fingerprint database. RSSI-based localization approaches have attempted to mitigate this radio-frequency (RF) phenomenon by using only LOS anchors for localization [13,14] or by filtering out unreliable signals based on calibrated thresholds [15]. Although effective, these binary approaches are limited when LOS paths are temporarily unavailable. Moreover, filtering solely based on calibrated thresholds could be unreliable when similar signal strengths correspond to vastly different channel conditions [5]. In large-scale indoor environments where anchor placement, environmental layout, and user mobility patterns are unknown, these limitations become more severe, resulting in lower localization accuracy [16,17].

Another major challenge is the high cost of constructing fingerprint maps, which typically requires labor-intensive site surveys and ground truth (x,y) positions. Supervised learning methods rely heavily on labeled data, making them sensitive to signal fluctuations, device heterogeneity, environmental dynamics, and they often require frequent retraining [18]. A further challenge is that binary LOS/NLOS classification does not explicitly represent partially obstructed propagation. In this study, LOS denotes an unobstructed direct path, PLOS represents partial obstruction, and NLOS denotes complete obstruction of the direct path. Following [19], PLOS is retained as an intermediate propagation category. To address these challenges, this study develops a confidence-adaptive BLE localization framework that does not require a surveyed position-fingerprint database at runtime. The framework combines three RSSI-derived indicators: relative anchor ranking, probabilistic LOS/PLOS/NLOS classification, and the instantaneous proportion of anchors whose predicted LOS probability exceeds a threshold. These indicators are integrated into a scan-level confidence score that adaptively determines the measurement-noise covariance of a Kalman filter applied to WCL position estimates. Unlike anchor-level reliability weighting, the proposed confidence formulation controls the filter’s trust in the aggregate WCL position without modifying individual WCL anchor weights. Although WCL, propagation classification, and Kalman filtering are established techniques, this study investigates their integration through the proposed RSSI-derived confidence formulation and evaluates its performance under different indoor propagation conditions.

The main contributions can be summarized as follows:

1. A scan-level confidence formulation combining relative RSSI ranking, probabilistic LOS/PLOS/NLOS reliability, and the instantaneous proportion of high-confidence LOS predictions to adapt the Kalman measurement covariance assigned to WCL position observations.
2. An evaluation of the intermediate PLOS category through an identical-protocol comparison of three-class classification with two binary alternatives, in which PLOS is merged with either LOS or NLOS.
3. An experimental evaluation against conventional WCL, a fixed-covariance Kalman filter, and single-indicator configurations in four environments, including two unseen zones and a high-traffic lobby.

The remainder of this paper is organized as follows. Section 2 reviews related work. Section 3 presents the problem formulation, the proposed methodology, and the experimental setup. Section 4 reports the results. Section 5 discusses key findings and limitations. Finally, Section 6 provides concluding remarks and directions for future work.

## 2. Related Work

Wireless signals such as Wi-Fi and BLE have been used extensively for indoor positioning. Although BLE dominates for cost-effective solutions, research on BLE localization specifically addressing BLE signal filtering or classification remains limited [7]. In many cases, methods of Wi-Fi RSS filtering have been adapted to BLE, as the same RSSI-based processing principles can give useful insights despite differences in signal behavior. Weighted centroid localization (WCL) estimates receiver position using known anchor coordinates weighted by RSSI-derived distance estimates [13]. Its computational simplicity and lack of a surveyed position-fingerprint database motivate its selection in this study. However, multipath propagation can distort the relative anchor weights, motivating the proposed confidence-adaptive refinement. Representative related approaches are summarized in Table 1.

### 2.1. Recursive Bayesian and Robust Kalman Filtering Approaches

Daníş et al. [26] proposed an adaptive Sequential Monte Carlo filter that dynamically adjusts the number of particles based on tracking uncertainty using BLE RSSI. The approach assumes a reliable motion model; sudden movement or rapid environmental changes can destabilize RSSI measurements, reducing accuracy in dynamic indoor environments. Qian et al. [27] proposed an improved particle filter combined with machine learning-based BLE fingerprinting to reduce indoor location estimation errors. Their method applies a particle filter over a pre-built fingerprint map in small enclosed environments, achieving good accuracy in rooms with dense anchor coverage and strong RSSI gradients; however, the zero-mean random-walk motion model limits scalability to large-scale environments such as long corridors and long trajectories, where particle diffusion cannot adequately cover the full trajectory. Robust Kalman filtering has also been investigated for measurement errors that deviate from conventional Gaussian noise assumptions. Jia et al. [28] proposed a Normal–Bernoulli-based robust Kalman filter for non-stationary heavy-tailed measurement noise. Their approach explicitly models the measurement-noise distribution, whereas the present study retains a conventional Kalman filter and adjusts its measurement covariance using confidence estimated from BLE RSSI observations. Consequently, the proposed approach does not explicitly model heavy-tailed measurement noise. Jia and Zhang [29] proposed a Normal–Gamma robust Kalman filter for MEMS/UWB indoor localization under NLOS conditions and reported errors of approximately 0.2 m. This value is given for context only: our BLE RSSI framework achieved MAEs of 1.85–4.06 m, but the sensing technologies, environments, and evaluation protocols differ, so the values are not directly comparable.

### 2.2. Fingerprint-Based and Landmark-Assisted Approaches

Junoh and Pyun [13] introduced a semi-crowdsourced indoor localization framework that used BLE landmarks [30] to label Wi-Fi fingerprints for the offline phase and used generative adversarial networks (GANs) [31] to generate a dense fingerprint map of an indoor area. While reducing data collection effort, the method assumes a fixed, known BLE anchor layout and predefined clusters focusing only on LOS anchors, limiting scalability to unseen environments with unknown layouts and variable RSSI propagation. Ai et al. [32] addressed anomalies in Wi-Fi RSSI fingerprints caused by human movement and wireless interference by automatically identifying abnormal fingerprints using an autoencoder; this approach improves system reliability but focuses on anomaly detection rather than adapting the radio map itself, and its effectiveness may decrease with previously unseen anomaly patterns. Xu et al. [12] investigated sparse RSS fingerprinting for visible-light positioning, while Zhou et al. [33] combined Wi-Fi positioning and inertial odometry through an extended Kalman filter. Unlike these approaches, the present framework uses BLE RSSI and known beacon coordinates without requiring a surveyed position-fingerprint database at runtime or additional inertial measurements.

### 2.3. Anchor Configuration and Sensor-Fusion Approaches

Sadowski and Spachos [7] optimized the number of BLE anchors for controlled LOS environments, assuming static geometry and labeled positions. Mahdavi et al. [34] improved RSS-based localization through an optimized fusion of multiple neural networks, demonstrating the benefits of combining complementary deep learning models for position estimation. Saghafi et al. [14] demonstrated a healthcare monitoring system using multiple wearable BLE transmitters and Raspberry Pi receivers, relying on trilateration. None of these three studies explicitly analyzes NLOS propagation effects, limiting their applicability to environments where LOS conditions cannot be assumed throughout the deployment.

### 2.4. LOS/NLOS-Aware Localization

Propagation-aware localization has been studied extensively for UWB, where channel impulse response features support reliable LOS/NLOS identification, whereas commodity BLE receivers generally expose only RSSI. Talebian et al. [20] proposed a lightweight binary LOS/NLOS detector for BLE direction finding based on constant tone extension IQ features; measurements classified as NLOS are discarded, and BLE 5.1 antenna-array hardware is required. An RSSI-based BLE system integrating LoS/NLoS classification into localization has also been reported [21], although it was evaluated only at short transmitter–receiver distances. Han et al. [8] combined BLE RSSI with probabilistic LOS/NLOS filtering and pedestrian dead reckoning, at the cost of additional inertial sensing. For Wi-Fi, Carpi et al. [15] detected and corrected NLOS RSSI measurements using tuned thresholds, while RTT-based methods [10,11,23] achieve higher accuracy but require ranging hardware and, in [10], prior knowledge of wall positions. Recent BLE studies have also addressed RSSI unreliability without propagation-state modeling. Pinto et al. [35] proposed OPTIMAPS, a site-survey-free system that determines log-distance model parameters from environment geometry and signal diversity and estimates position by sequential least-squares optimization; like the present work, it avoids a fingerprint database, but it does not distinguish LOS and NLOS observations. Kumar and Bhutani [36] applied DBSCAN clustering to remove outlying RSSI readings before federated Kalman filtering, reporting a 13% error reduction. In contrast, the proposed framework retains all observations and adapts the Kalman measurement covariance according to RSSI-derived propagation confidence. Table 1 compares the approaches most closely related to this work. In the table, "static" denotes data collected at fixed reference points with a stationary device, and "moving" denotes measurements obtained during user motion, either stop-and-go or continuous walking. These approaches differ from the proposed framework mainly in where reliability information enters the estimator. Hard anchor-level decisions, such as NLOS discarding or correction [15,20] and LOS-only WCL [13,14], exclude or modify anchors before positioning and become unreliable when a path is only partially obstructed.

Robust Kalman filters [28,29] model the measurement-noise distribution explicitly, adaptive Kalman filters estimate noise covariances from the data [37], and fixed-covariance Kalman filtering [7,38] applies the same trust to every observation. The proposed framework instead derives the measurement covariance of each WCL observation from soft three-class LOS/PLOS/NLOS probabilities, relative RSSI ranking, and the proportion of high-confidence LOS predictions, without excluding anchors and without inertial sensing, ranging hardware, or a fingerprint database. Table 2 further summarizes the practical challenges addressed, namely labeled-data requirements, adaptability to layout changes, and robustness to anchor ordering.

## 3. Materials and Methods

### 3.1. Problem Formulation

**Figure 1:**
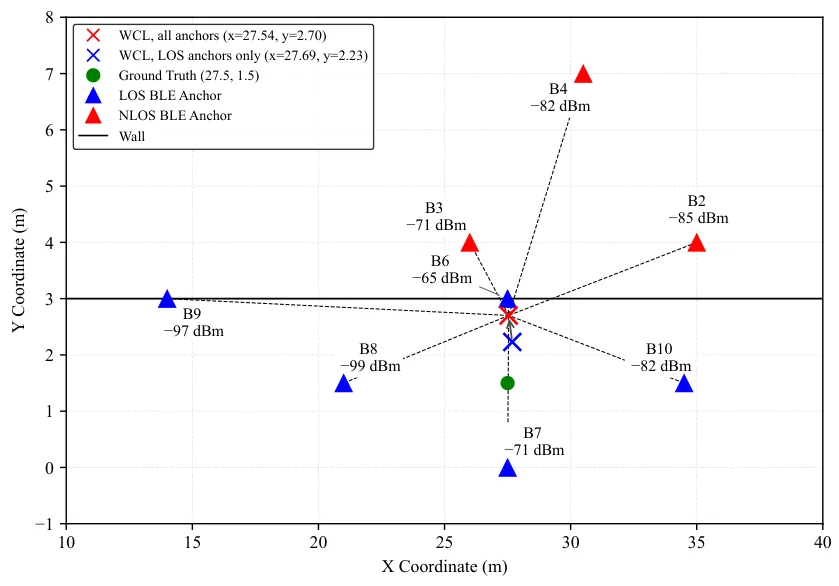
Effect of NLOS anchors on the WCL estimate for the reference position (27.5, 1.5) m. WCL using all visible anchors estimates (27.54, 2.70) m (error 1.20 m), whereas WCL using only the LOS anchors estimates (27.69, 2.23) m (error 0.75 m). The LOS-only estimate assumes known propagation states and serves as an illustrative reference; it is not available during online localization.

BLE RSSI-based indoor localization is affected by signal attenuation, reflection, and multipath propagation caused by walls, furniture, and other obstructions [5,40]. Consequently, RSSI measurements obtained under LOS and NLOS conditions may overlap, as illustrated in Figure 1. This ambiguity introduces errors into RSSI-based distance estimation and can degrade localization accuracy. Weighted centroid localization (WCL) estimates the receiver position from the known coordinates of BLE anchors and their RSSI-derived weights [13].

The distance between the receiver and anchor *i* is approximated using the log-distance path-loss model:

(1) $$d_{i}=10^{\frac{A-{\mathrm{RSSI}}_{i}}{10n}},$$

where $d_{i}$ is the estimated distance between the receiver and anchor *i*, *A* denotes the reference RSSI at 1 m and *n* is the path-loss exponent. The exponent depends on the propagation environment, and its uncertainty can affect RSSI-based distance estimation [41]. In this study, a fixed value of n=2.5 is adopted following the BLE-WCL configuration in [13]. Using a common exponent avoids environment-specific or anchor-specific parameter adjustment, although it does not eliminate propagation-model mismatch. The inverse-square distance weights are defined as

(2) $$w_{i}=\frac{1}{d_{i}^{2}},$$

and the receiver position is estimated as

(3) $$\hat{x}=\frac{\sum_{i=1}^{N}w_{i}x_{i}}{\sum_{i=1}^{N}w_{i}},\;\hat{y}=\frac{\sum_{i=1}^{N}w_{i}y_{i}}{\sum_{i=1}^{N}w_{i}},$$

where $\left(x_{i},y_{i}\right)$ represents the coordinates of anchor *i*, and *N* is the number of anchors used for localization. Substituting Equation (1) into Equation (2), the relative weights of two anchors satisfy

(4) $$\frac{w_{i}}{w_{j}}=10^{\frac{{\mathrm{RSSI}}_{i}-{\mathrm{RSSI}}_{j}}{5n}}.$$

Thus, the common reference RSSI cancels from the relative weights, whereas the path-loss exponent controls their relative magnitudes. A smaller *n* concentrates the weights on the strongest, possibly NLOS, anchors, whereas a larger *n* spreads them toward the geometric centroid. Unlike multilateration, which estimates position from geometric constraints imposed by individual range measurements, WCL combines anchor coordinates through normalized weights. This provides a computationally simple localization approach but does not eliminate errors caused by unreliable RSSI measurements. In multipath-affected environments, stronger RSSI does not necessarily indicate a shorter propagation distance. Consequently, NLOS anchors may receive disproportionately large weights. Figure 1 illustrates this effect for a single scan at the reference position (27.5, 1.5) m. The NLOS anchors B3 and B4, located behind the wall, are received at −71 and −82 dBm, the same levels as the LOS anchors B7 and B10. WCL therefore assigns them comparable weights despite the obstruction. Using all visible anchors, WCL estimates (27.54, 2.70) m (error 1.20 m), whereas using only the LOS anchors yields (27.69, 2.23) m (error 0.75 m). The NLOS anchors thus shift the estimate toward the obstructed region and increase the error by 0.45 m.

**Figure 2:**
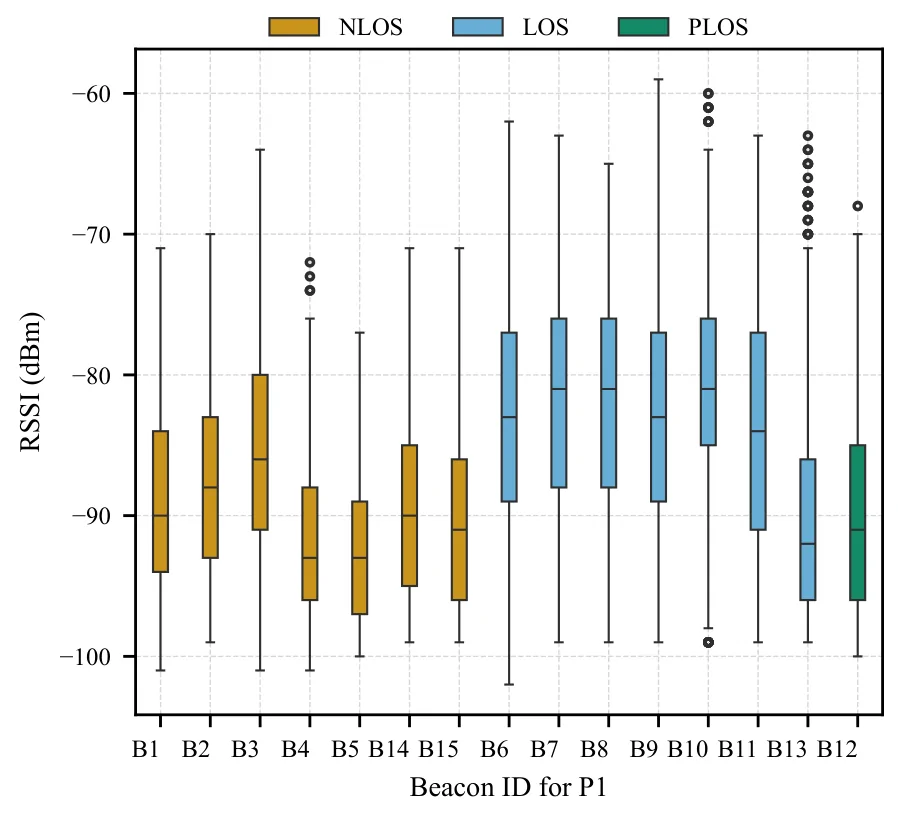
Representative per-anchor RSSI distribution by propagation class (Zone 1, Path 1). The boxplots show the median, interquartile range, and whiskers; black circles indicate outlier observations. Several NLOS anchors overlap with LOS anchors in RSSI, and some LOS anchors show consistently weak signal, so the propagation classes cannot be separated by RSSI level alone. The same overlap is observed in the other zones.

In practical deployments, propagation labels are not available at runtime, so the LOS-only estimate cannot be computed directly. Because an NLOS anchor can be received at the same RSSI level as a LOS anchor, as shown in Figure 1 and for all anchors in Figure 2, a single RSSI value cannot reliably distinguish LOS from NLOS measurements. The proposed framework therefore does not attempt hard LOS/NLOS separation or exclude anchors. Propagation labels are used only offline, to train the classifier (Section 3.2.2). During online localization, the trained classifier estimates LOS/PLOS/NLOS probabilities from temporal RSSI statistics and relative rank without any labels, and these probabilities adjust the Kalman filter’s trust in each WCL observation (Section 3.2.3).

### 3.2. Methodology

**Figure 3:**
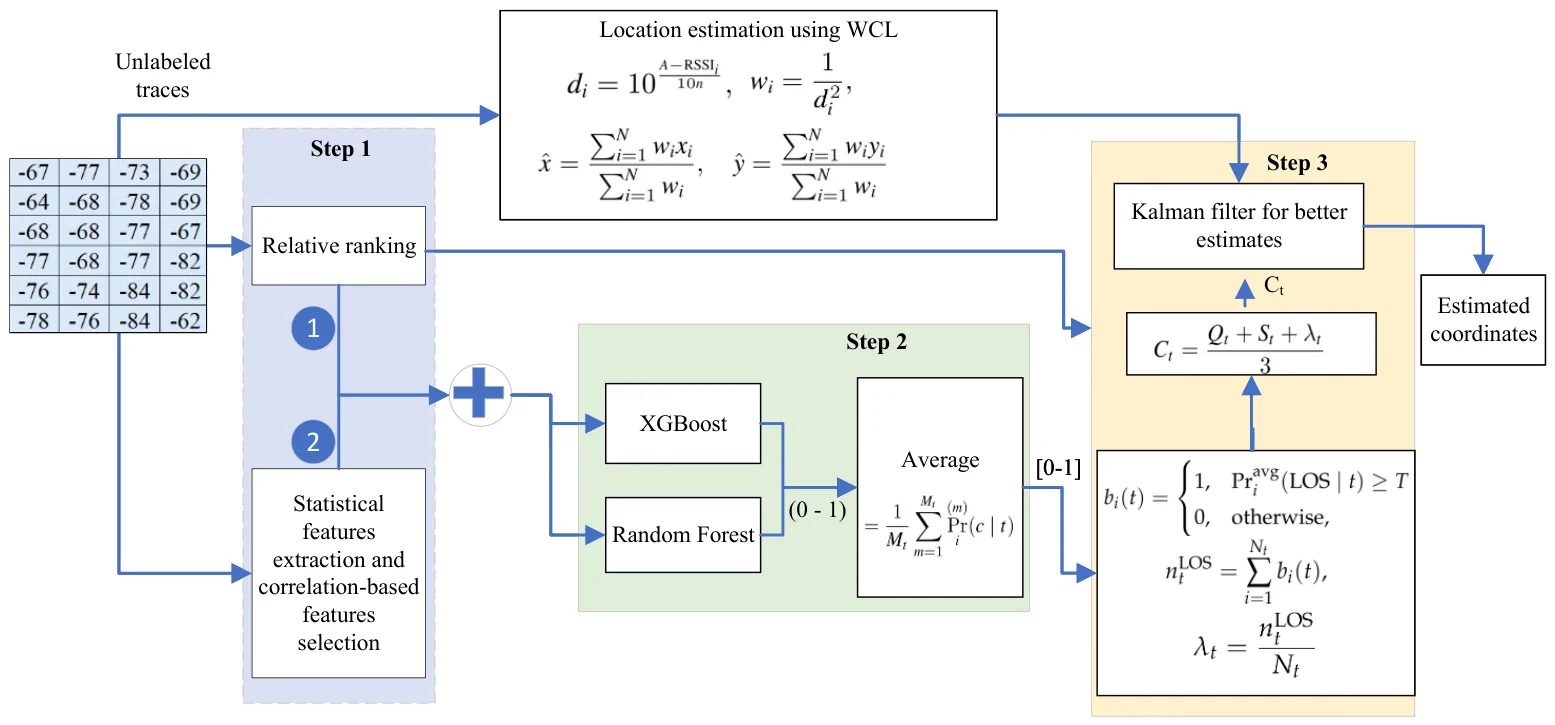
Stepwise workflow of the proposed BLE localization system: (Step 1) relative ranking of BLE anchors and feature extraction, (Step 2) Propagation channel classification, (Step 3) confidence-score-based Kalman filtering, and output estimated coordinates.

This section presents the proposed BLE-based indoor localization framework. Figure 3 presents the overall workflow of the proposed confidence-adaptive indoor positioning framework. The process starts with multiple BLE signal traces collected on each path, processed through the three steps described below.

#### 3.2.1. Step 1: Feature Extraction

A dual feature-extraction strategy combines relative ranking and statistical signal features. In the relative-ranking stream (① in Figure 3), the RSSI values of all visible BLE anchors in each scan are ordered from strongest to weakest, assigning rank 1 to the strongest anchor and a fixed penalty rank, one beyond the largest attainable rank for the anchor pool (rank 20 for the 19-anchor pool shared by Zones 1–4; rank 15 for the 14-anchor lobby deployment), to anchors not observed in the current scan, so that absent or weak anchors receive a consistent low-reliability penalty. The rank score $Q_{t}\in \left[0,1\right]$ is computed as the rank-based weighted average of normalized RSSI values, in which anchors are weighted in inverse proportion to their rank, so that top-ranked anchors contribute more strongly to $Q_{t}$ than lower-ranked anchors. Formally, let $r_{i,t}$ be the rank of anchor *i* at time *t* (rank 1 = strongest; the penalty rank for unobserved anchors), and let ${\mathrm{RSSI}}_{i,t}\in \left[0,1\right]$ be its min–max-normalized RSSI over the anchor pool. The rank score is the rank-weighted mean of normalized RSSI,

(5) $$Q_{t}=\frac{\sum_{i=1}^{N_{t}}\;r_{i,t}^{-1}\;{\mathrm{RSSI}}_{i,t}}{\sum_{i=1}^{N_{t}}\;r_{i,t}^{-1}},$$

so that top-ranked anchors dominate the average and $Q_{t}$ is high only when highly ranked anchors also carry strong normalized RSSI. All visible BLE anchors in each scan are retained for WCL; no fixed-size subset of the strongest anchors is selected. RSSI ranking is used to calculate the rank-based confidence indicator, not to exclude anchors. Consequently, the number of anchors contributing to WCL varies with signal availability.

The second stream (② in Figure 3) computes statistical features from each anchor’s RSSI trace using a sliding window of 10 consecutive timestamps, capturing temporal signal behavior that complements the instantaneous rank score. A Pearson correlation matrix, with threshold |Correlation|>τ, τ=0.84, is used to remove redundant features. Of the 12 candidate features, three were excluded (maximum RSSI, RSSI range, and Unique RSSI count), and the following nine were retained:

- Mean and standard deviation: average RSSI level and dispersion.
- Skewness and kurtosis: asymmetry and peakedness of the RSSI distribution.
- Median absolute deviation (MAD): outlier-robust measure of dispersion.
- Interquartile range (IQR): spread of the middle 50% of RSSI values.
- RSSI slope: short-term increasing or decreasing trend across scans.
- Minimum RSSI: weakest RSSI value within the window.
- Flatness ratio (Flat_ratio): ratio of unique RSSI values to window size, showing how much the signal fluctuates in corresponding sliding window.

Combining multiple statistical features rather than relying on a single one has also been shown to improve indoor localization [42]. Features from both streams are concatenated into a single vector, forming the input to the classification models described in Step 2.

#### 3.2.2. Step 2: Propagation Channel Classification Using an Ensemble Model

The Propagation channel of each BLE anchor is modeled with a soft classification into three classes, LOS, PLOS, and NLOS, using an ensemble of two complementary tree-based models:

- Random Forest [43]: a bagging-based model that reduces variance and overfitting on unseen data when applied to statistical RSSI features and relative ranking.
- XGBoost [44,45]: a gradient boosting model that captures complex feature interactions across LOS, PLOS, and NLOS signals in noisy or previously unseen environments.

Two additional architectures, an attention-based network for tabular data (TabNet [46]) and a recurrent model capturing temporal dependencies across sliding windows (LSTM [47,48]), were also evaluated but not retained in the deployed ensemble (Section 4). Because LOS observations substantially outnumber PLOS and NLOS ones, the Synthetic Minority Over-sampling Technique (SMOTE) [49] (default k=5 nearest neighbors) was applied to the training partition only, resampling PLOS and NLOS up to the LOS count; validation and test trajectories were left untouched to avoid leakage. Each retained model m∈{1,…,M} outputs, for anchor *i* at time *t*, a three-class probability vector,

(6) $$\overset{\left(m\right)}{\underset{i}{\mathrm{Pr}}}\left(t\right)=\left[\begin{array}{c} {\mathrm{Pr}}_{i}^{\left(m\right)}\left(\mathrm{LOS}|t\right)\\ {\mathrm{Pr}}_{i}^{\left(m\right)}\left(\mathrm{PLOS}|t\right)\\ {\mathrm{Pr}}_{i}^{\left(m\right)}\left(\mathrm{NLOS}|t\right) \end{array}\right],$$

satisfying ${\mathrm{Pr}}_{i}^{\left(m\right)}\left(\mathrm{LOS}|t\right)+{\mathrm{Pr}}_{i}^{\left(m\right)}\left(\mathrm{PLOS}|t\right)+{\mathrm{Pr}}_{i}^{\left(m\right)}\left(\mathrm{NLOS}|t\right)=1$. To reduce model-specific uncertainty, the final soft probability for anchor *i* and class *c* is obtained by class-wise averaging across the ensemble,

(7) $$\overset{\mathrm{avg}}{\underset{i}{\mathrm{Pr}}}\left(c|t\right)=\frac{1}{M}\sum_{m=1}^{M}\overset{\left(m\right)}{\underset{i}{\mathrm{Pr}}}\left(c|t\right),\;c\in \left\{\mathrm{LOS},\mathrm{PLOS},\mathrm{NLOS}\right\},$$

where M=2 is the ensemble size (Random Forest and XGBoost). This averaging, performed element-wise per class, reduces the impact of individual model bias and allows the same anchor to transition between LOS, PLOS, and NLOS states as the tag device moves.

#### 3.2.3. Step 3: Applying an Adaptive Kalman Filter with a Confidence Score

The position estimated by WCL is refined using a Kalman filter with a constant-velocity motion model. The measurement-noise covariance $R_{t}$ determines the relative influence of the predicted state and the current position observation [37].

To account for variations in the estimated reliability of WCL observations, the proposed framework combines three RSSI-derived indicators into a scan-level confidence score.

The combined confidence is defined as

(8) $$C_{t}=\frac{Q_{t}+S_{t}+\lambda_{t}}{3},$$

where $Q_{t}$ is the relative RSSI-ranking score defined in Section 3.2.1, $S_{t}$ represents the estimated propagation reliability, and $\lambda_{t}$ denotes the proportion of high-confidence LOS predictions.

For each available anchor *i*, the ensemble class probabilities from Equation (7) are converted into a reliability score:

(9) $$\begin{array}{cc} s_{i,t}= & \alpha \;\overset{\mathrm{avg}}{\underset{i}{\mathrm{Pr}}}\left(\mathrm{LOS}|t\right)\\ \; & +\beta \;\overset{\mathrm{avg}}{\underset{i}{\mathrm{Pr}}}\left(\mathrm{PLOS}|t\right)\\ \; & +\gamma \;\overset{\mathrm{avg}}{\underset{i}{\mathrm{Pr}}}\left(\mathrm{NLOS}|t\right). \end{array}$$

The coefficients (α,β,γ)=(1.0,0.5,0.1) encode the assumed reliability ordering LOS > PLOS > NLOS. They are fixed design parameters rather than empirically optimized values. Consequently, their relative magnitudes represent a heuristic reliability model, not calibrated propagation probabilities.

The propagation-reliability score is averaged over the $N_{t}$ available anchors:

(10) $$S_{t}=\frac{1}{N_{t}}\sum_{i=1}^{N_{t}}s_{i,t},\;N_{t}>0.$$

The third indicator, $\lambda_{t}$, measures the proportion of available anchors whose predicted LOS probability reaches the fixed threshold T=0.8. This threshold was selected heuristically to identify relatively high-confidence LOS predictions. Its empirical optimality and sensitivity have not been established. The binary indicator is defined as

(11) $$b_{i}\left(t\right)=\left\{\begin{array}{cc} 1, & {\mathrm{Pr}}_{i}^{\mathrm{avg}}\left(\mathrm{LOS}|t\right)\geq T,\\ 0, & \mathrm{otherwise}. \end{array}\right.$$

The resulting high-confidence LOS proportion is

(12) $$\lambda_{t}=\frac{1}{N_{t}}\sum_{i=1}^{N_{t}}b_{i}\left(t\right).$$

The threshold identifies anchors with relatively high predicted LOS probability, rather than guaranteeing their actual propagation states. Unlike temporal stability measures, this indicator describes the high-confidence LOS proportion within the current observation.

The three indicators are combined with equal weights to avoid introducing additional fitted coefficients. Although they summarize different aspects of the observations, the propagation-reliability score and high-confidence LOS proportion are both derived from the predicted class probabilities and are therefore not statistically independent.

The confidence score controls the Kalman measurement covariance:

(13) $$R_{t}=C_{t}R_{\min}+\left(1-C_{t}\right)R_{\max},$$

where $R_{\min}=\sigma_{\min}^{2}I$ and $R_{\max}=\sigma_{\max}^{2}I$, with $0<\sigma_{\min}^{2}\leq \sigma_{\max}^{2}$. In this study, $\sigma_{\min}^{2}=0.1$ m^2^ and $\sigma_{\max}^{2}=10$ m^2^. Only these two values were tuned, on Zone 1–2 training trajectories, and they were applied unchanged to all other environments. The resulting $\sigma_{\max}\approx 3.2$ m is of the same order as the spread of conventional WCL errors on those trajectories (Std 1.92 m; 90% of errors below 4.48 m; Section 4.2).

For the specified reliability coefficients, the anchor-level scores lie between 0.1 and 1.0. Assuming the three predicted class probabilities sum to one and the ranking score lies between zero and one, the combined confidence is bounded between 0.1/3 and 1.

Consequently, the adapted covariance satisfies

(14) $$R_{\min}⪯R_{t}⪯R_{\max}.$$

This formulation provides bounded measurement-covariance adaptation. Higher estimated confidence assigns a covariance closer to the specified minimum, increasing the relative influence of the WCL observation. Lower confidence produces a covariance closer to the specified maximum, reducing its influence on the Kalman filter update. The confidence score is calculated once per observation and adjusts the covariance of the aggregate WCL position estimate. It does not modify the individual RSSI-derived anchor weights used in WCL. Table 3 illustrates the expected filtering behavior under different confidence configurations.

A two-dimensional constant-velocity Kalman filter is implemented with the state vector

(15) $$x_{t}={\left[x_{t},y_{t},{\dot{x}}_{t},{\dot{y}}_{t}\right]}^{T},$$

with process noise $Q_{w}=\mathrm{diag}\left(0.01,0.01,0.01,0.01\right)$.

The innovation is the difference between the WCL position observation $z_{t}$ and its predicted value. Each path is processed independently: the filter state is initialized from the first WCL observation of each path and the MAE is then computed per path.

#### 3.2.4. Estimated Location Output

The output of the proposed approach is a sequence of estimated (x,y) coordinates for unlabeled trajectories, referenced to the known anchor coordinate system. The term “unlabeled” refers specifically to the absence of surveyed (x,y) reference-point coordinates at runtime and does not imply the absence of all supervision. The ensemble classifier is trained offline using static, anchor-level LOS/PLOS/NLOS labels (Section 3.4), and per-sample ground truth for evaluation is obtained by interpolation (Section 3.3). No surveyed position labels are required at inference time, which is the sense in which the runtime method is label-free. The method is therefore label-free at inference but not training-free. It performs online propagation-state prediction, not verified online labeling: it does not establish ground-truth propagation states or update its labels, and it requires known anchor coordinates and a trained classifier. Although it was evaluated in unseen zones, deployment in fully unmapped sites has not been established.

### 3.3. Experimental Setup

Localization experiments were conducted in several indoor settings to evaluate the effect of RSSI classification and relative rank information on location estimation in previously unseen environments.

#### Data Collection

**Figure 4:**
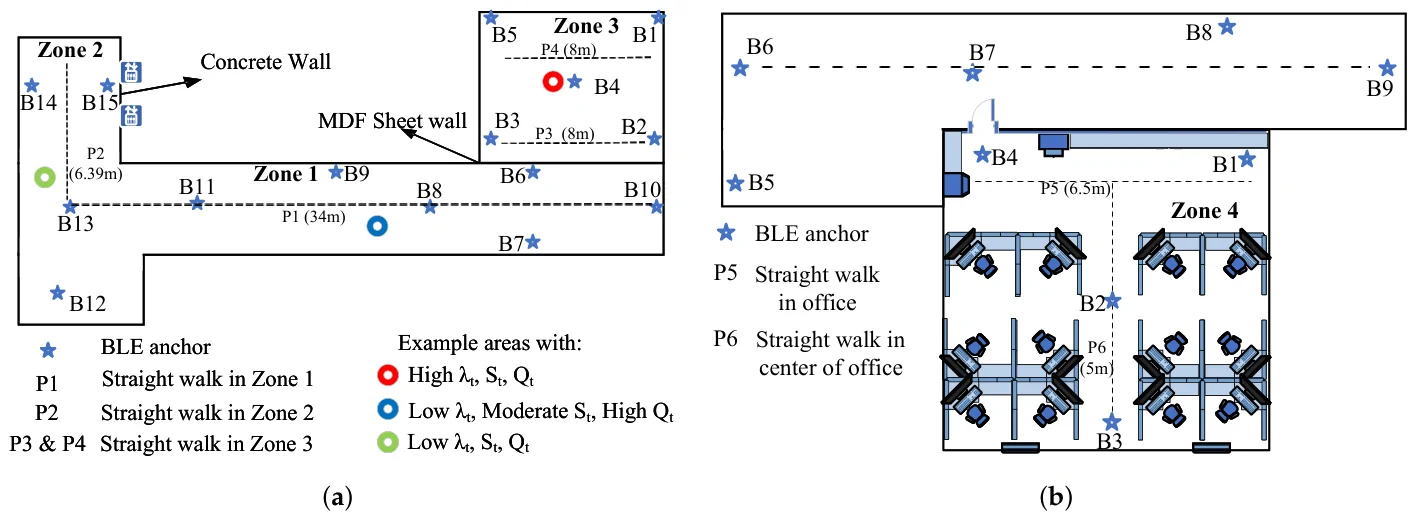

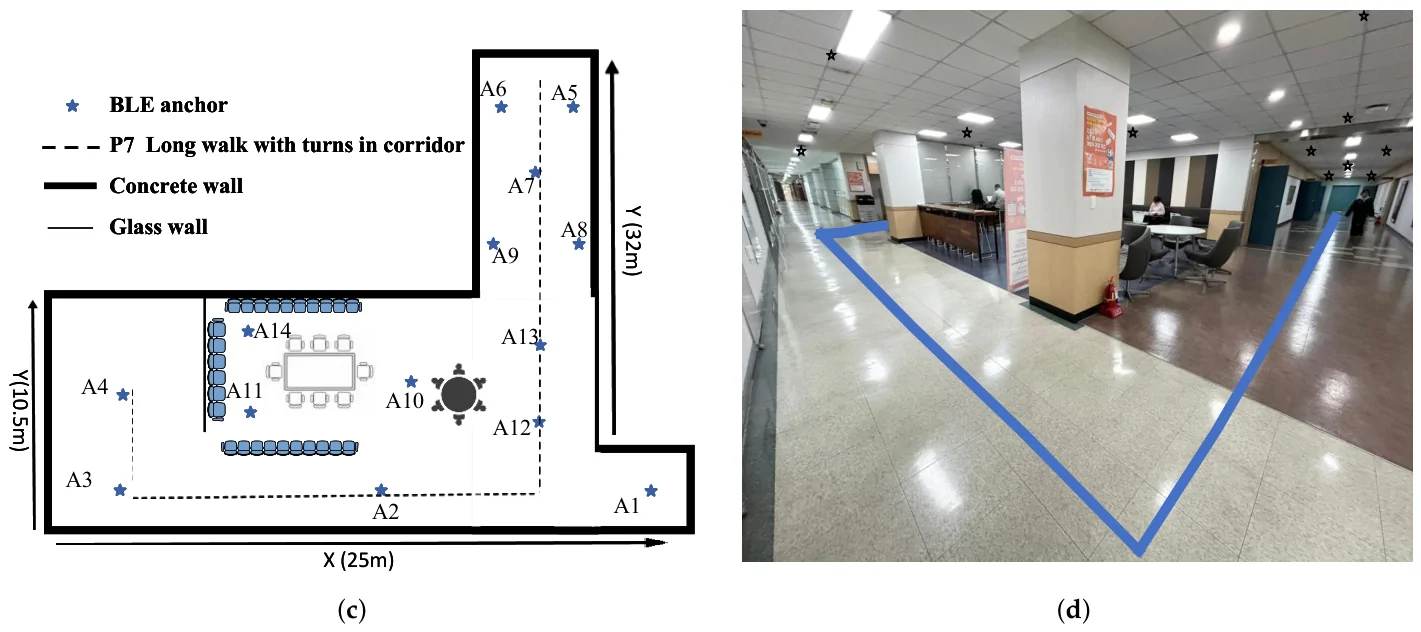
Experimental environments and BLE anchor deployment. (**a**) Zones 1–3, including Corridor I, Corridor II (front of the elevator), and the classroom. Circular markers indicate three representative environmental scenarios used to illustrate the operational logic of $C_{t}$ in Table 3. (**b**) Zone 4, including the office room and Corridor I. (**c**) Expanded experimental area comprising the lobby and connecting corridors with anchor deployment and pedestrian traffic as Path P7 on second floor. (**d**) Physical view of the expanded experimental area Path P7.

BLE signal traces were collected using a custom Android application deployed on two smartphones, a Samsung Galaxy Note8 (Samsung Electronics, Suwon, Republic of Korea) and a Redmi Note9 (Xiaomi, Beijing, China) with Android 9 and Android 10, respectively, across corridors, classrooms, and office environments on the eighth floor of the IT building at Chosun University. RSSI acquisition is a standard BLE function on such widely available handsets, which is consistent with the commodity-hardware scope of this work. The transmission power of all anchors was set to −4 dBm with a broadcasting interval of 300 ms, and each scan recorded RSSI from all visible anchors approximately once per second, producing a matrix of RSSI values with associated timestamps. An RSSI value of −200 dBm was assigned to unobserved signals to flag missing packets. This value is only a marker for absence and is dropped before computing the statistical features and the min–max normalization, so missing readings do not skew the feature values. Reference positions for localization evaluation were obtained by linear interpolation between the known endpoints of each walking path under a constant-speed assumption. The interpolated coordinates provide a common reference for comparing localization methods evaluated on identical trajectories. However, they are not independently measured receiver positions at every timestamp. Unrecorded variations in walking speed may therefore introduce reference-position uncertainty. The floor layout for experiments is illustrated in Figure 4.

An additional experiment was conducted in the lobby adjacent to the classrooms on the second floor of the same building to evaluate the proposed framework under highly dynamic conditions. This environment experienced substantial pedestrian traffic, ranging from approximately 10 to 15 people during normal periods to 20 to 40 people during class transitions, introducing frequent signal fluctuation, human body shadowing, and dynamic LOS/NLOS transitions that provide a demanding testbed for assessing localization robustness.

### 3.4. Low-Traffic Testbed Setup

BLE RSSI is collected in four zones:

- Zone 1 (P1): Corridor I area (35 m × 2.5 m) with 7 LOS, 7 NLOS, and 1 PLOS anchor.
- Zone 2 (P2): Corridor II (10.5 m × 3 m) elevator front area containing 4 LOS, 5 NLOS, and 6 PLOS anchors.
- Zone 3 (P3, P4): Classroom (10 m × 8 m) with 5 LOS, 3 NLOS, and 2 PLOS anchors.
- Zone 4 (P5, P6): Office room (9 m × 7 m) with 4 LOS and 7 NLOS anchors and 1 PLOS anchor.

Zones 1–2 are used for classifier training and covariance tuning, whereas Zones 3–4 are unseen environments (Section 4). Zones 1–4 are mutually adjacent regions served by a shared pool of 19 physical BLE anchors; each zone observes only a subset of this pool, so a given anchor may present as LOS along one path and NLOS along another, depending on the walking geometry. The per-zone LOS/PLOS/NLOS counts listed above refer to the anchors observed within each zone and are therefore overlapping subsets of the 19-anchor pool, not disjoint sets. Data collection in these four zones took place during the summer break period, when pedestrian activity in the building was minimal, providing a comparatively stable propagation environment relative to the high-traffic testbed described in Section 3.5. LOS, NLOS, and PLOS labels were assigned offline through manual inspection of the architectural floor plan and the relative geometry of the anchors and walking paths.

An anchor was labeled LOS when no structural obstacle obstructed its direct path to the walking area, NLOS when a concrete or MDF wall obstructed the direct path, and PLOS under partially obstructed conditions, including glass partitions and partially open doorways. These labels were assigned at the anchor–zone level rather than for each individual receiver position, since RSSI samples were collected continuously while the surveyor walked along the path. Consequently, the labeling procedure approximates the propagation conditions within each zone and cannot represent every temporary obstruction or transition occurring during movement.

The assigned labels provide supervision exclusively during offline classifier training. During deployment, propagation-state probabilities are estimated from the observed RSSI-derived features without requiring predefined LOS/PLOS/NLOS labels or surveyed receiver positions for each measurement. Because the training labels remain fixed within each anchor–zone combination, the classifier may learn characteristics associated with anchor placement in addition to propagation-dependent RSSI patterns. This limitation may affect generalization when the receiver geometry or obstruction conditions differ from those represented during training.

The proposed method incorporates the predicted class probabilities into scan-level measurement confidence instead of relying exclusively on hard propagation-state decisions. Nevertheless, probabilistic predictions cannot eliminate systematic errors introduced by coarse reference labeling.

Anchor deployment layouts are shown in Figure 4a for Zones 1 through 3 and in Figure 4b for Zone 4, with the high-traffic deployment shown in Figure 4c. Zone 3 provides a near-ideal deployment for WCL, whereas in Zone 1 and Zone 2 anchors are placed primarily along the walking corridor, so that localization performance depends strongly on anchor placement, signal geometry, and LOS/NLOS conditions.

Across all four zones, the per-anchor RSSI distributions overlap between propagation classes: NLOS anchors can resemble LOS anchors because of multipath reflection in narrow corridors and off metallic surfaces, LOS anchors can appear weak under temporary human obstruction, and PLOS anchors show wide, inconsistent variance. This overlap, illustrated for Zone 1 in Figure 2 confirms that physical layout and obstruction cannot be reliably separated using hard RSSI thresholds, which motivates the soft-probability channel classification and rank feature used in the proposed framework.

### 3.5. Expanded and High Traffic Testbed Setup

A further experiment was conducted in a larger environment consisting of a 32 m by 4 m corridor connected to an 11 m by 8 m lobby with concrete and glass walls as illustrated in Figure 4c. Fourteen BLE anchors were deployed with spacing of 3 to 7 m in the lobby and 6 to 10 m along the corridors. A single continuous walking trajectory was recorded, beginning at one point, passing through two turning points, and ending at another, forming a multi-directional path with straight segments and turns across both regions, walked without stopping to produce a realistic, smoothly varying motion pattern shown in Figure 4d.

## 4. Performance Evaluation

**Figure 5:**
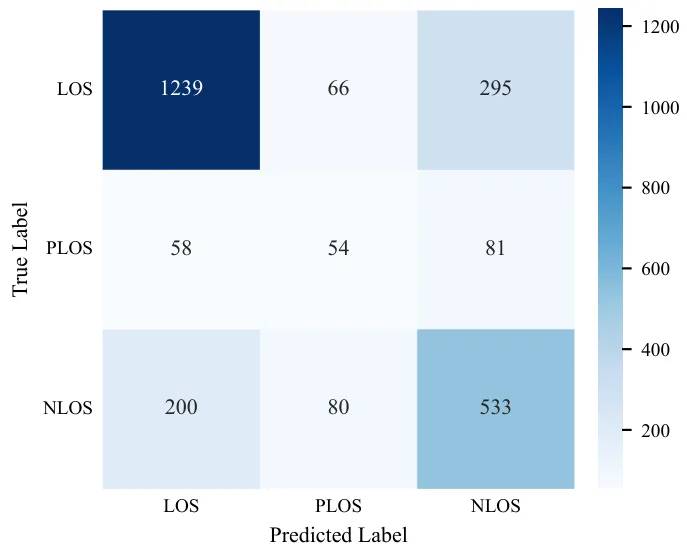
Confusion matrix of LOS, PLOS, and NLOS classification on the unseen test set from Zones 3 and 4.

The proposed system consists of two major stages: propagation channel classification using an ensemble of Random Forest and XGBoost, and location estimation using WCL, refined through the confidence-adaptive Kalman filter. The same preprocessing steps used for training are applied to the test data before prediction and location refinement. Experiments were conducted across the four zones described in Section 3.4 (Paths P1 to P6), together with an evaluation in the expanded and high-traffic area described in Section 3.5 (Path P7), covering both corridor and lobby regions under a continuous, multi-directional walking trajectory with turning. The ensemble classifier is trained exclusively on data from Zones 1 and 2 and evaluated on the unseen Zones 3 and 4 (Figure 5). The covariance bounds $\sigma_{\min}^{2}$ and $\sigma_{\max}^{2}$ are likewise tuned only on Zone 1–2 trajectories. Consequently, the corridor results (Zones 1–2) are in-domain, whereas the classroom (Zone 3), office (Zone 4), and high-traffic path P7 are out-of-domain, exposed to neither classifier training nor covariance tuning.

### 4.1. Propagation Channel Classification Performance

For the classification stage, we evaluated four models, Random Forest, XGBoost, TabNet, and LSTM, using selected statistical features with correlation threshold (τ=0.84) and relative ranking. Of these, the two tree-based models, Random Forest and XGBoost, generalized best to unseen environments (Table 4) and were retained as the deployed ensemble, whereas TabNet and LSTM were excluded owing to weaker generalization and substantially higher computational cost.

The key training parameters and complexity of all four candidate models used for RF channel classification are summarized in Table 5. The tree-based models, i.e., Random Forest and XGBoost, are relatively simple with minimal tuning and short training times. In contrast, TabNet and LSTM took significantly longer to train. To avoid overfitting, early stopping is applied to all models except Random Forest. The LSTM model is trained using 5-fold cross-validation. From Table 4, it can be observed that even though the classification models performed well on training data, their accuracy decreased when applied to new, unseen environments. Confusion matrix in Figure 5 reflects the challenges of generalizing classification using only statistical features and rank. It can be seen that the ensemble performs well on LOS signals, correctly identifying 1239 of 1600 samples, with the remainder misclassified as PLOS or NLOS. However, PLOS is more difficult to classify, with only 54 of 193 correct predictions, because of the overlap of PLOS with LOS and NLOS shown in box-plot Figure 2. For NLOS, the ensemble achieves moderate accuracy, correctly identifying 533 of 813 samples. The misclassifications of 200 NLOS samples as LOS and 80 as PLOS may be due to strong reflected signals.

Class imbalance was addressed using the Synthetic Minority Over-sampling Technique (SMOTE), applied exclusively to the training partition. The validation and test partitions retained their original class distributions. As shown in Table 6, overall classification accuracy may conceal poor performance on minority class, class-wise precision, recall, F1-score, macro-F1 (0.56), and balanced accuracy (0.57) are reported alongside overall accuracy (0.70). The ensemble achieves an F1-score of 0.80 for LOS and 0.62 for NLOS, whereas PLOS achieves only 0.27. The lower PLOS performance indicates difficulty distinguishing partially obstructed observations from the other propagation categories. Although SMOTE addresses the imbalance in the training data, it does not guarantee accurate discrimination of overlapping propagation classes. Consequently, the proposed framework uses the predicted class probabilities as one component of the scan-level confidence score rather than treating individual class predictions as error-free propagation labels.

Nevertheless, systematic classification errors may affect the estimated measurement confidence and subsequent localization performance.

### 4.2. Localization Performance

Localization performance was evaluated across the recorded trajectories using mean absolute error (MAE), standard deviation (Std), and 90th-percentile positioning error (P90).

The baseline comparisons were selected to examine the effects of confidence-based refinement relative to conventional WCL and fixed-covariance Kalman filtering. Conventional WCL establishes the performance of the underlying RSSI-based position estimator, while the fixed-covariance Kalman filter provides a reference for evaluating adaptive measurement covariance.

The additional configurations incorporating the individual confidence indicators provide partial ablation comparisons. These comparisons evaluate the selected configurations rather than establishing the independent contribution of every confidence component.

The evaluation is restricted to WCL-based configurations because the proposed framework specifically investigates confidence-adaptive refinement of WCL position observations. Comparisons with other non-fingerprint positioning algorithms are outside the scope of the present experiments.

- **Traditional WCL**: inverse-square RSSI weighting (Equations (1)–(3)) without channel awareness.
- **WCL with $Q_{t}$** and **WCL with $S_{t}$**: the adaptive filter of Step 3 with $C_{t}$ replaced by $Q_{t}$ or $S_{t}$ alone.
- **WCL + fixed-covariance KF**: the same Kalman filter with a constant R=diag(50,50), selected to minimize MAE on the Zone 1–2 training trajectories. This represents the fixed-covariance smoothing commonly used for BLE RSSI localization [7,38] and is the main baseline for the adaptive covariance.
- **Proposed**: the adaptive filter with $C_{t}$ from Equation (8) and the parameters given in Step 3.

Because $\lambda_{t}$ is not isolated in these configurations, its individual contribution cannot be quantified. Table 7 reports the results, including the change in MAE relative to traditional WCL.

**Figure 6:**
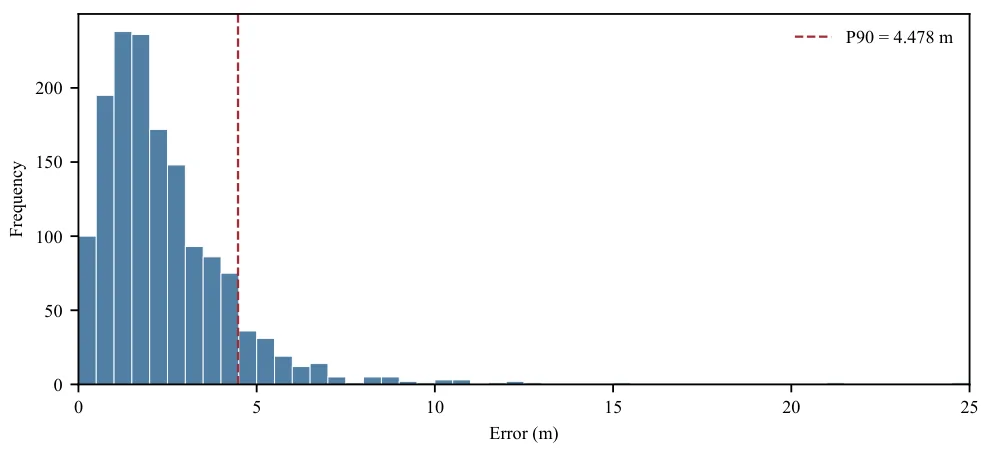
Distribution of WCL Euclidean positioning errors on Paths P1–P2 using the reference coordinates. The dashed line indicates the 90th percentile.

Figure 6 shows the distribution of conventional WCL errors on the training corridors. Most errors are below 5 m (P90 = 4.478 m), but the distribution contains occasional large positioning deviations.

All configurations reduced MAE relative to traditional WCL in every environment, and the proposed method achieved the lowest MAE and P90 in all four. In the in-domain corridors, MAE decreased from 2.391 m to 2.042 m (14.6%) and P90 from 4.478 m to 3.855 m. In the unseen classroom, MAE decreased from 2.037 m to 1.848 m (9.3%) and P90 from 3.615 m to 3.078 m (14.9%). In the unseen office, MAE decreased from 2.698 m to 2.608 m (3.3%), the smallest improvement.

**Figure 7:**
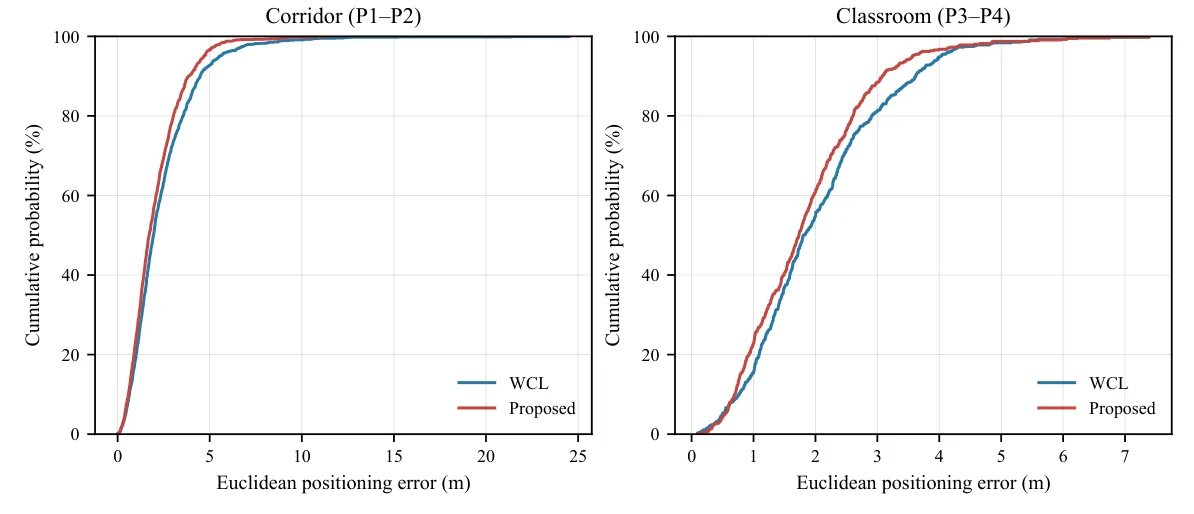
CDFs of Euclidean positioning errors for conventional WCL and the proposed method in the corridor (P1–P2) and classroom (P3–P4).

**Figure 8:**
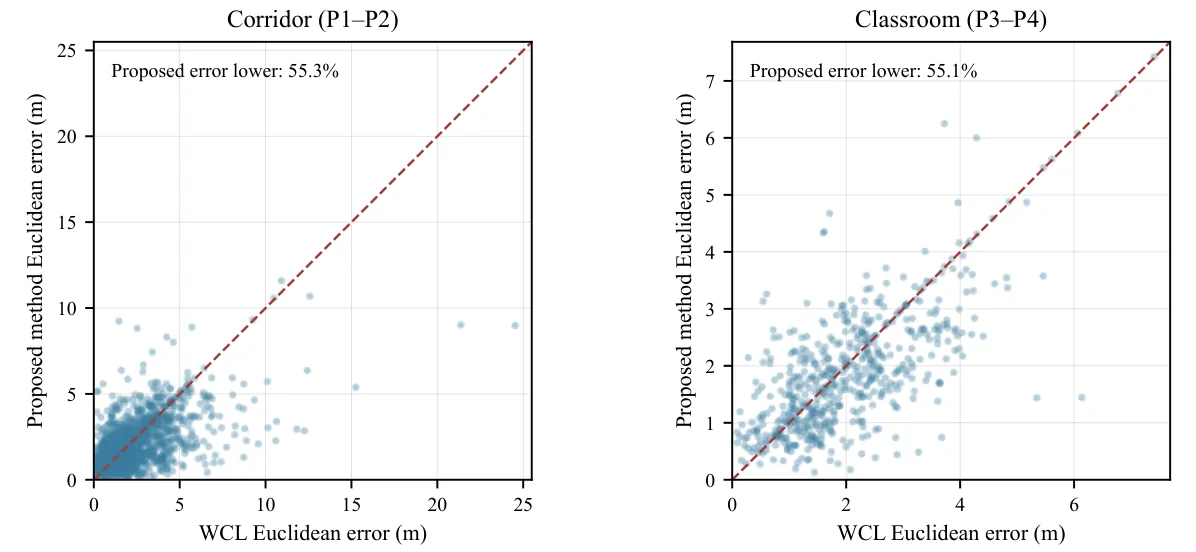
Paired comparison of per-scan Euclidean positioning errors for conventional WCL and the proposed method in the corridor (P1–P2) and classroom (P3–P4). Each point represents the errors for the same scan; the dashed line indicates equal errors.

Figure 7 shows the CDFs of the positioning error for the corridor (P1–P2) and the classroom (P3–P4), representing an extended corridor and a smaller indoor area. The curves lie close together at small errors and separate in the upper part of the distribution, consistent with the lower P90 values reported above. Figure 8 compares the paired per-scan errors. Over all scans, the proposed error is lower for 55.3% of scans in the corridor and 55.1% in the classroom, but the improvement is concentrated in the large-error region: among the scans whose WCL error exceeds the WCL P90, the proposed error is lower in 86% and 73% of cases, and the mean error in this subset decreases from 6.47 to 3.71 m and from 4.32 to 3.42 m, respectively. In the corridor, the number of errors exceeding 5 m decreases from 108 to 50. The proposed method therefore mainly suppresses large, NLOS-induced deviations rather than improving every individual scan. Results for all evaluated environments are reported in Table 7.

Temporal filtering accounts for most of the gain in the low-traffic zones: the fixed-covariance filter alone reduced MAE by 12.8%, 8.6%, and 2.9% in the corridors, classroom, and office. The additional reduction from adaptive covariance was 0.042 m (2.0%), 0.013 m (0.7%), and 0.011 m (0.4%), respectively. These differences are small relative to plausible reference-position errors from interpolation (Section 5) and should not be interpreted as conclusive. The single-indicator configurations gave smaller reductions (2.9–5.5%) than the proposed method in all three zones, and smaller than the fixed-covariance filter in the corridor and classroom.

In the office, the Std (approximately 3.7 m) exceeded the MAE for every configuration while P90 stayed below 4 m, indicating that a small number of large-error observations dominate the dispersion. In these scans, the strongest anchor was located at (34, 10.5) m, outside the office, with an RSSI of −69 dBm, and other strong anchors lay in the same distant region. The raw and merged RSSI records agree for this recording, so the errors reflect received signal conditions rather than data processing. Because WCL weights depend only on RSSI, these scans pull the estimate toward the distant anchors; the confidence score does not modify anchor weights and can only reduce the influence of such observations in the filter update.

**Figure 9:**
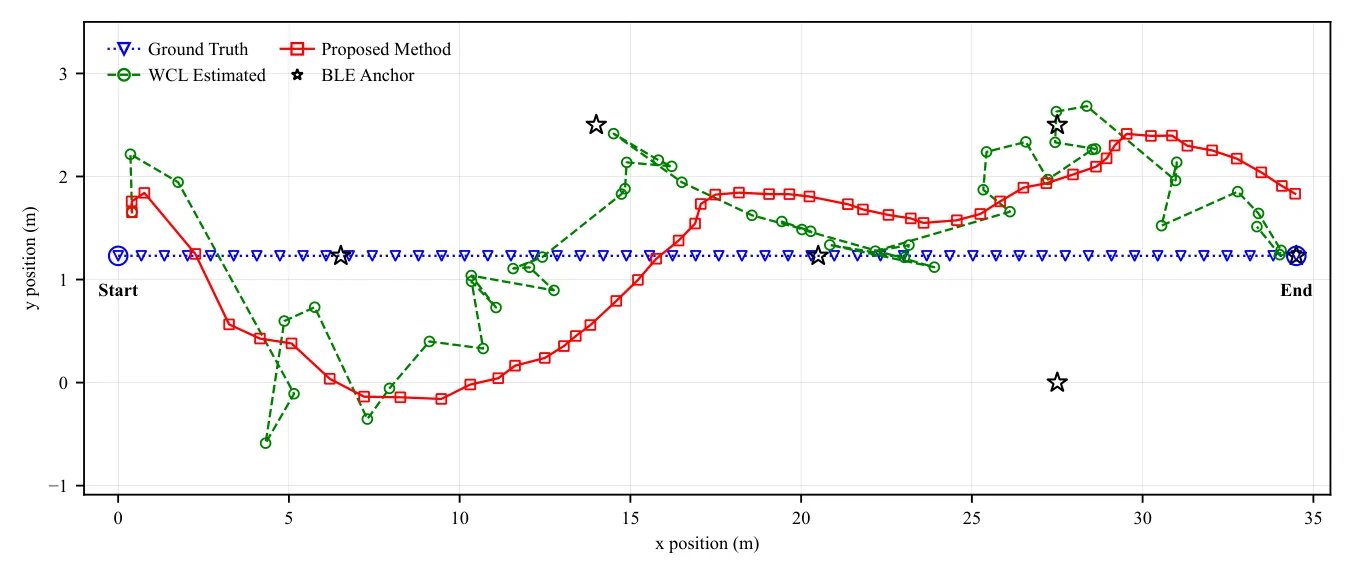
Ground-truth trajectory, traditional WCL estimates, and the proposed confidence-adaptive-Kalman WCL estimates for the first traversal of Path P1. Point markers denote individual position estimates, and lines represent the corresponding trajectories.

Figure 9 illustrates the trajectory estimation for a mobile tag in Zone 1. Traditional WCL produces irregular estimates with large deviations near NLOS anchors along the corridor walls. The proposed method suppresses these deviations and produces a smoother trajectory; however, because the filter relies partly on the motion-model prediction, it can lag behind the true motion and locally increase the error where the WCL estimate is already accurate.

### 4.3. Evaluation on Expanded and High-Traffic Testbed

**Figure 10:**
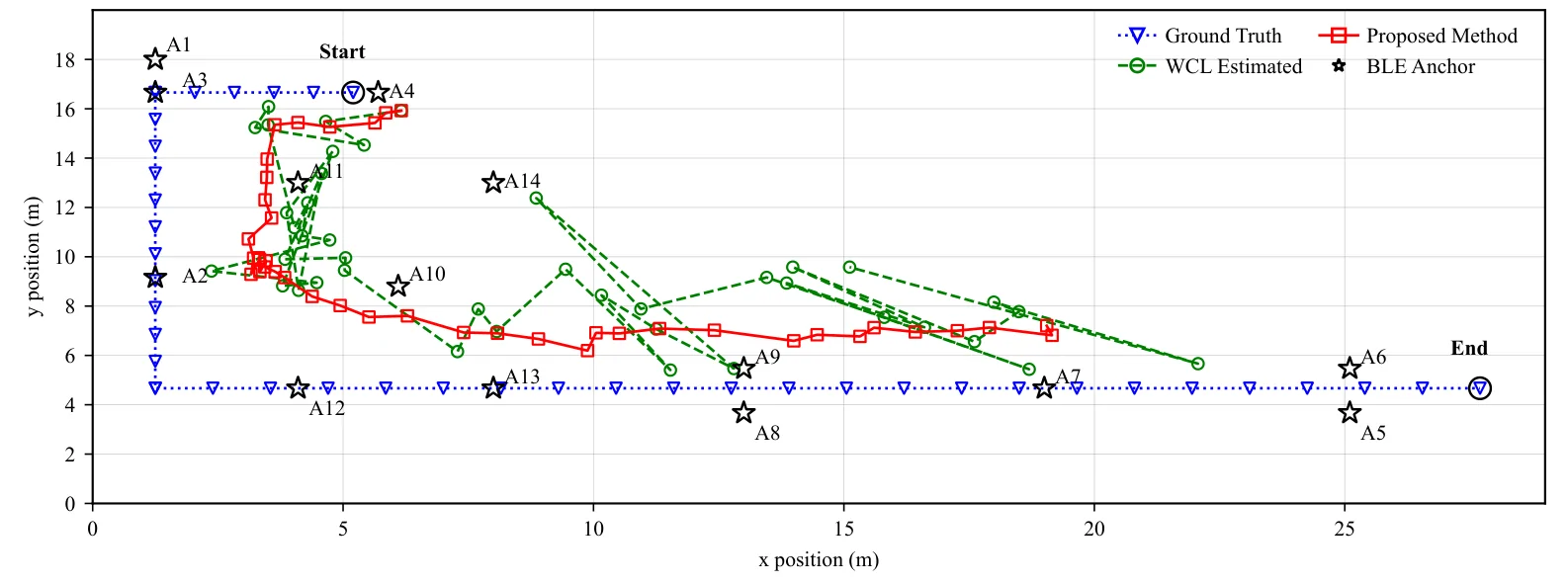
Ground-truth trajectory, traditional WCL estimates, and proposed confidence-adaptive-Kalman WCL estimates for the first traversal of Path P7 in the lobby environment. Point markers denote individual position estimates, and lines represent the corresponding trajectories.

On the high-traffic path P7, the proposed method reduced MAE from 4.408 m to 4.059 m (7.9%), Std from 3.346 m to 3.124 m, and P90 from 7.995 m to 7.684 m. The fixed-covariance filter, whose covariance was selected on low-traffic corridor data, reduced MAE by only 0.2% and slightly increased P90 (8.003 m). Using $Q_{t}$ or $S_{t}$ alone reduced MAE by 5.2% and 6.3%. P7 is therefore the environment where adapting the covariance per scan provided the clearest benefit over a fixed covariance (7.7% lower MAE), consistent with the time-varying propagation caused by body shadowing and orientation changes under heavy pedestrian traffic. Figure 10 shows the first traversal of P7. Traditional WCL drifts near the turns, where the dominant anchors switch rapidly between LOS and NLOS. The proposed trajectory also deviates transiently at the turns because the constant-velocity motion model briefly lags the change in direction; these deviations are short-lived and have limited effect on the MAE. A coordinated-turn or IMU-aided motion model could reduce them. All channel states in these experiments were predicted at runtime from RSSI features; no propagation state was known in advance.

### 4.4. Three-Class Versus Binary Formulation

To evaluate the contribution of the PLOS category, two binary classifiers were trained by merging PLOS with either NLOS or LOS. All three configurations were evaluated using identical test observations, preprocessing procedures, and localization settings to isolate the effect of the propagation-class formulation.

In Table 6, the three-class ensemble achieved an overall accuracy of 70%, with a macro-F1 of 0.56 and a balanced accuracy of 0.57 on unseen dataset. The PLOS class remained difficult to classify, with precision, recall, and F1 scores of 0.27, 0.28, and 0.27, respectively. To determine whether retaining PLOS as a separate class still provides useful information for localization, the proposed three-class formulation was directly compared with the two binary formulations.

As shown in Table 8, the proposed three-class formulation reduced the localization error compared with both binary formulations across the evaluated environments. Compared with the LOS versus PLOS+NLOS formulation, the three-class method reduced the error by 39.0%, 11.2%, and 7.4% in Zones 1–2, Zone 3, and Zone 4, respectively. Compared with the NLOS versus PLOS+LOS formulation, the corresponding reductions were 39.2%, 11.4%, and 7.6%. These results do not imply that PLOS is classified reliably, but they indicate that retaining PLOS as a separate intermediate category can provide useful information for the localization stage compared with the evaluated binary formulations.

## 5. Discussion

Several limitations bound the scope of the reported results, particularly regarding the estimation method, parameterization, and experimental evaluation.

The proposed pipeline uses two tree-based models, Random Forest and XGBoost, while excluding the more computationally demanding TabNet and LSTM models. In the low-traffic zones, a well-tuned fixed-covariance Kalman filter achieved most of the improvement; the clearest benefit of adaptation appeared under high pedestrian traffic, where the fixed covariance tuned on corridor data did not transfer. The confidence score adjusts the filter’s trust in the aggregate WCL observation but not the anchor weights, so scans dominated by strong signals from distant anchors, as observed in the office, are not corrected. The inference procedure consists of two tree-based predictions and one Kalman update per scan at approximately 1 Hz; however, on-device latency and energy consumption have not been measured.

Regarding parameterization, only $\sigma_{\min}^{2}$ and $\sigma_{\max}^{2}$ were tuned, on Zone 1–2 trajectories, and applied unchanged to the remaining environments. The path-loss exponent *n*, the coefficients α, β, γ, the threshold *T*, and the equal weighting in $C_{t}$ were fixed by design, and $S_{t}$ and $\lambda_{t}$ are statistically dependent. Their sensitivity and environment-dependent optimization remain unexplored.

Reference positions were obtained by constant-speed interpolation between known trajectory endpoints. Evaluation methodologies distinguish surveyed reference points from independently measured trajectories [50], and independent references are particularly important for dynamic systems [51]. Because intermediate positions were not measured, interpolation uncertainty could not be quantified, and small differences between methods, such as those between the proposed method and the fixed-covariance filter in Zones 1–4, need to be interpreted cautiously. The evaluation follows common reporting practice for indoor positioning, including mean and 90th-percentile errors over multiple zones and walking paths, but it is not claimed to comply with ISO/IEC 18305 [52], which additionally requires surveyed reference points of substantially higher accuracy than the system under test.

The static per-anchor labels reflect anchor placement as well as propagation conditions and cannot capture time-varying LOS/NLOS transitions. Although SMOTE was applied to the training partition and balanced accuracy and macro-F1 are reported, PLOS remains poorly separated, and residual classification errors may propagate into the confidence score.

Future work will investigate robust and non-Gaussian estimators such as heavy-tailed Kalman filters and particle filters; anchor-level down-weighting of implausible distant anchors; coordinated-turn or IMU-aided motion models; learned or environment-dependent weighting of $Q_{t}$, $S_{t}$, and $\lambda_{t}$; sensitivity analysis of the fixed parameters; on-device latency and energy profiling; independently measured reference trajectories; per-sample propagation labels; and cross-building and cross-device validation.

## 6. Conclusions

In this study, we proposed a confidence-adaptive Kalman refinement of WCL for BLE indoor localization that requires no surveyed reference-point labels at runtime. A scan-level confidence score combining relative RSSI ranking, probabilistic LOS/PLOS/NLOS classification, and the proportion of high-confidence LOS predictions sets the measurement covariance assigned to each WCL position observation. Relative to conventional WCL, the method reduced MAE by 14.6% in corridors, 9.3% in a classroom, 3.3% in an office, and 7.9% on a high-traffic lobby trajectory, with the lowest MAE and P90 among the evaluated configurations in every environment. Paired per-scan analysis showed that the improvement is concentrated in the large-error region, where most NLOS-induced deviations occur, rather than uniformly across all scans. In the low-traffic zones, most of this gain is also obtained by a fixed-covariance Kalman filter, and the additional benefit of adaptation is small; under high pedestrian traffic, the adaptive covariance reduced MAE by 7.7% relative to the fixed-covariance filter. Because of the proposed method’s modular design, it can be extended in the future with an IMU and a non-Gaussian estimator, such as a particle filter operating directly on the confidence-weighted measurement. Domain adaptation and cross-building, cross-device validation are further directions for future work.

## Figures and Tables

**Table 1:** Comparison of LOS/NLOS-aware and RSSI-based indoor localization approaches most closely related to this work.

Paper	Algorithm/Method	Static/Moving	LOS/NLOS Evaluation	Key Findings and Limitations
[20] (2026)	BLE AoA direction finding; binary LOS/NLOS detection from CTE IQ features using Nyström kernel approximation with SVC, compared with RF and MLP	Static	Yes (binary)	Kernel feature mapping improved detection accuracy by about 7–14% over linear baselines. NLOS channels are identified and discarded. The method requires BLE 5.1 antenna-array hardware and was evaluated with a controlled artificial blocker rather than natural building obstructions.
[21] (2025)	BLE RSSI localization with an ML-based LoS/NLoS classification module and feedback process for activity monitoring	Static	Yes (binary)	Integrates channel-condition classification into RSSI-based localization. Evaluation was limited to short transmitter–receiver distances (0.5–4 m) with static and moving human obstacles, not continuous walking trajectories across rooms.
[10] (2025)	Wi-Fi RTT with AP visibility matching and a multi-wall model	Static/Moving	Yes	Reduced positioning error from 2.3 m to 0.64 m using NLOS-aware AP visibility. Requires RTT-capable hardware, a known environment, and prior wall-position information.
[11] (2024)	Hybrid Wi-Fi RTT and RSS fingerprinting with Gaussian filtering and an LOS/NLOS classifier	Moving	Yes	The labeled RTT and RSS combination improved accuracy by up to 50% compared with RSS-only methods. However, reliance on labeled LOS/NLOS data may limit generalization to unseen environments.
[8] (2018)	BLE RSSI and pedestrian dead-reckoning with probabilistic LOS/NLOS filtering	Moving with PDR	Yes	Combining RSSI, PDR, and LOS/NLOS information improved positioning accuracy, but the method depends on additional inertial sensing.
[22] (2022)	BLE RSSI with mean and median preprocessing followed by an HMM and particle filter	Moving	No	Robust statistical preprocessing before particle filtering. Filter parameters were calibrated under controlled conditions, which may reduce adaptability when obstacles or human movement alter propagation paths.
[23] (2024)	LOS/NLOS detection from RTT and RSS using Random Forest, LS-SVM, and a deep neural network	Moving	Yes	Achieved at least 96% classification accuracy using only 10 samples. However, the method was not extensively evaluated under dynamically changing environmental conditions.
[15] (2023)	Wi-Fi RSSI localization with LOS/NLOS detection and NLOS correction	Static	Yes	NLOS-aware correction reduced positioning error by up to 70%. The approach requires threshold tuning for NLOS detection and may not adapt well across different indoor environments.
[24] (2023)	Comparison of BLE RSSI fingerprinting with a multi-carrier phase-difference method using manual calibration and wKNN	Static	No	Requires specialized hardware for phase measurements and antenna/receiver calibration. Phase ambiguity and multipath can degrade performance.
[25] (2021)	BLE RSSI-based fingerprinting and trilateration using anchors installed at 1.8 m height	Static	No	Evaluated using manually labeled training data. Receiver orientation and human-body shadowing were not explicitly modeled.

**Table 2:** Comparison of Challenges Addressed by the Proposed Approach.

Challenge	Existing/Traditional Methods	Proposed Approach
Data-label requirement [18,26,39]	Supervised fingerprinting approaches require large collections of precisely labeled (x,y) ground-truth positions for training.	Refines WCL-based estimates using probabilistic LOS classification, rank features, and Kalman filtering, thereby reducing dependence on labeled position data.
Adaptability [38,39]	Models trained directly on RSSI values often overfit to a specific anchor deployment and lose localization accuracy when anchors are moved or the layout changes.	Uses rank scores and per-anchor statistical features to estimate LOS probabilities, reducing dependence on a fixed anchor layout within the evaluated environments.
Anchor-order dependence [13]	Methods commonly assume a fixed anchor set and a consistent ordering of RSSI inputs, making them sensitive to anchor addition, removal, or reordering.	Introduces an anchor-order-invariant representation by extracting statistical features independently for each anchor, allowing adaptation to addition, removal, and reordering of anchors.

**Table 3:** Environmental scenario examples and adaptive response of the confidence-adaptive Kalman filter.

Environment Scenario	$Q_{t}$	$S_{t}$	$\lambda_{t}$	$C_{t}$	Filter Response
Ideal LOS environment	↑	↑	↑	↑	$R_{t}\approx R_{\min}$ (Trust WCL more)
Strong Multipath environment	↑	∼	↓	∼	$R_{\min}<R_{t}<R_{\max}$ (Trust WCL less)
Weak/Noisy environment	↓	↓	↓	↓	$R_{t}\approx R_{\max}$ (Trust prediction more)

Symbols: ↑ = high, ↓ = low, ∼ = moderate.

**Table 4:** Propagation channel classification accuracy (%) per model.

Model	Train	Unseen
Random Forest	87.2	71.19
XGBoost	88.1	68.32
TabNet	85.4	59.1
LSTM	75.0	63.5

**Table 5:** Configuration of the machine learning and deep learning models evaluated for channel classification. Only Random Forest and XGBoost are retained in the deployed ensemble.

Parameter	RF	XGBoost	TabNet	LSTM
Trees/layers	100	300	$N_{d}=8,N_{a}=8$, 3 steps	2 layers (64 + 32)
Maximum depth	Unlimited	6	–	–
Learning rate	–	0.1	–	–
Optimizer	–	–	Adam	Adam
Batch size	–	–	256	128
Dropout	–	–	–	0.3
Sequence length	–	–	–	10 steps
Objective/loss	–	Softmax	Softmax (3)	Cross-entropy
Early stopping	No	20 epochs	20 epochs	5 epochs
Cross-validation	No	No	No	k=5
Training time	∼1 min	∼3 min	∼8 min	∼15 min

**Table 6:** Per-class performance of the RF+XGB ensemble on the unseen test set.

Class	Precision	Recall	F1	Support
LOS	0.83	0.77	0.80	1600
PLOS	0.27	0.28	0.27	193
NLOS	0.59	0.66	0.62	813
Accuracy		0.70 (2606 samples)		
Macro-F1		0.56		
Balanced accuracy		0.57		

**Table 7:** Localization performance per environment. ΔMAE is the percentage MAE reduction relative to Traditional WCL; positive values indicate improvement.

Environment	Method	Anchors	MAE (m)	ΔMAE (%)	Std (m)	P90 (m)
Corridor (Zones 1–2) Paths P1–P2	Traditional WCL	15	2.391	–	1.917	4.478
	WCL with $Q_{t}$		2.278	4.73	1.849	4.247
	WCL with $S_{t}$		2.278	4.73	1.842	4.237
	WCL with fixed covariance KF		2.084	12.84	1.473	3.924
	Proposed Method		2.042	14.60	1.421	3.855
Classroom (Zone 3) Paths P3–P4	Traditional WCL	10	2.037	–	1.129	3.615
	WCL with $Q_{t}$		1.926	5.45	1.048	3.194
	WCL with $S_{t}$		1.928	5.35	1.052	3.194
	WCL with fixed covariance KF		1.861	8.64	1.031	3.108
	Proposed Method		1.848	9.28	1.043	3.078
Office (Zone 4) Paths P5–P6	Traditional WCL	12	2.698	–	3.713	3.840
	WCL with $Q_{t}$		2.614	3.11	3.719	3.562
	WCL with $S_{t}$		2.620	2.89	3.716	3.562
	WCL with fixed covariance KF		2.619	2.93	3.701	3.529
	Proposed Method		2.608	3.34	3.690	3.522
Expanded High-Traffic Area, Path P7	Traditional WCL	14	4.408	–	3.346	7.995
	WCL with $Q_{t}$		4.179	5.20	3.347	7.794
	WCL with $S_{t}$		4.129	6.33	3.368	7.771
	WCL with fixed covariance KF		4.397	0.25	3.202	8.003
	Proposed Method		4.059	7.92	3.124	7.684

**Table 8:** Localization MAE (m) of the three-class model and two binary variants in which PLOS is merged with LOS or NLOS.

Model	Zones 1–2	Zone 3	Zone 4
Traditional WCL	2.391	2.037	2.698
Three-class (proposed)	2.042	1.848	2.608
Binary (LOS vs. PLOS + NLOS)	3.350	2.082	2.815
Binary (NLOS vs. PLOS + LOS)	3.361	2.086	2.823

## Data Availability

Data is contained within the article.
